# “Immune TOR-opathies,” a Novel Disease Entity in Clinical Immunology

**DOI:** 10.3389/fimmu.2018.00966

**Published:** 2018-05-09

**Authors:** Sophie Jung, Laura Gámez-Díaz, Michele Proietti, Bodo Grimbacher

**Affiliations:** ^1^CNRS, UPR 3572 (I2CT), Institut de Biologie Moléculaire et Cellulaire (IBMC), Strasbourg, France; ^2^Hôpitaux Universitaires de Strasbourg, Pôle de Médecine et de Chirurgie Bucco-Dentaires, Strasbourg - Université de Strasbourg, Faculté de Chirurgie Dentaire, Strasbourg, France; ^3^Center for Chronic Immunodeficiency (CCI), Medical Center – Faculty of Medicine, University of Freiburg, Freiburg, Germany

**Keywords:** AKT, immune dysregulation, kinase, mTOR, PI3k, primary immunodeficiency, S6K

## Abstract

Primary immunodeficiencies (PIDs) represent a group of mostly monogenic disorders caused by loss- or gain-of-function mutations in over 340 known genes that lead to abnormalities in the development and/or the function of the immune system. However, mutations in different genes can affect the same cell-signaling pathway and result in overlapping clinical phenotypes. In particular, mutations in the genes encoding for members of the phosphoinositide3-kinase (PI3K)/AKT/mTOR/S6 kinase (S6K) signaling cascade or for molecules interacting with this pathway have been associated with different PIDs that are often characterized by the coexistence of both immune deficiency and autoimmunity. The serine/threonine kinase mechanistic/mammalian target of rapamycin (mTOR), which acts downstream of PI3K and AKT, is emerging as a key regulator of immune responses. It integrates a variety of signals from the microenvironment to control cell growth, proliferation, and metabolism. mTOR plays therefore a central role in the regulation of immune cells’ differentiation and functions. Here, we review the different PIDs that share an impairment of the PI3K/AKT/mTOR/S6K pathway and we propose to name them “immune TOR-opathies” by analogy with a group of neurological disorders that has been originally defined by PB Crino and that are due to aberrant mTOR signaling (1). A better understanding of the role played by this complex intracellular cascade in the pathophysiology of “immune TOR-opathies” is crucial to develop targeted therapies.

## Introduction

Primary immunodeficiencies (PIDs) comprise more than 350 inherited disorders that affect the development and/or the functions of the components of the immune system (2, 3). They are individually rare but collectively, they are “more common than thought” (4), particularly due to the rapid increase in the number of newly described disorders and of causative genes that have been identified. In fact, the study of PIDs has frequently contributed to the discovery of new genes that are pivotal in immune cell development, effector functions, or in the maintenance of immune homeostasis (5). Susceptibility to severe and recurrent infections is a constant clinical manifestation in PID patients. However, an overlap between immune deficiency (infections and/or malignancies) and immune dysregulation (autoimmunity, autoinflammation, and/or allergy) is often observed in certain types of PIDs (2, 3, 6). Although PIDs are mostly inherited as monogenic disorders, disease penetrance, as well as disease expressivity, may result from interactions between genetic, epigenetic, and/or environmental factors. This contributes to the wide phenotypic diversity, even between individuals with an identical mutation in the same gene (2, 3, 7). The International Union of Immunological Societies (IUIS) PID expert committee regularly publishes a classification based on shared pathogenesis and/or clinical phenotypes with the latest update in 2017 (2, 3).

The serine/threonine kinase mechanistic/mammalian target of rapamycin (mTOR) plays a central role within the phosphoinositide3-kinase (PI3K)/AKT/mTOR/S6 kinase (S6K) signaling pathway. It acts as a downstream effector of AKT in two structural and functional distinct protein complexes named mTOR complex 1 and 2 (mTORC1 and mTORC2, respectively) (8). mTOR integrates the different cues from the microenvironment to control cell growth, proliferation, and metabolism, thereby exerting crucial functions in the regulation of immune homeostasis (8, 9).

Defects in the genes encoding for the different members of the PI3K/AKT/mTOR/S6K cascade or for molecules interacting with this pathway are frequently associated with immune dysfunction. We therefore propose here to cluster the different PIDs that share an impairment of the PI3K/AKT/mTOR/S6K pathway. Considering the central role of mTOR in the signaling cascade, this subgroup of PIDs will be referred hereafter as “immune TOR-opathies.” The term “mTOR-opathies” was initially coined in 2007 by PB Crino to define a wide spectrum of neurological disorders due to abnormal mTOR signaling that are characterized by focal malformations of cortical development, epilepsy, and neurobehavioral disabilities (1, 10).

In this review, we describe the PI3K/AKT/mTOR/S6K signaling cascade, focusing on the genetic and molecular defects of the different “immune TOR-opathies,” and on the impact of this pivotal pathway in the development of immune deficiency and immune dysregulation, a hallmark of “immune TOR-opathies.”

## PI3K/AKT/mTOR/S6K Signaling Pathway Plays a Crucial Role in Immune Homeostasis

**Figure 1:**
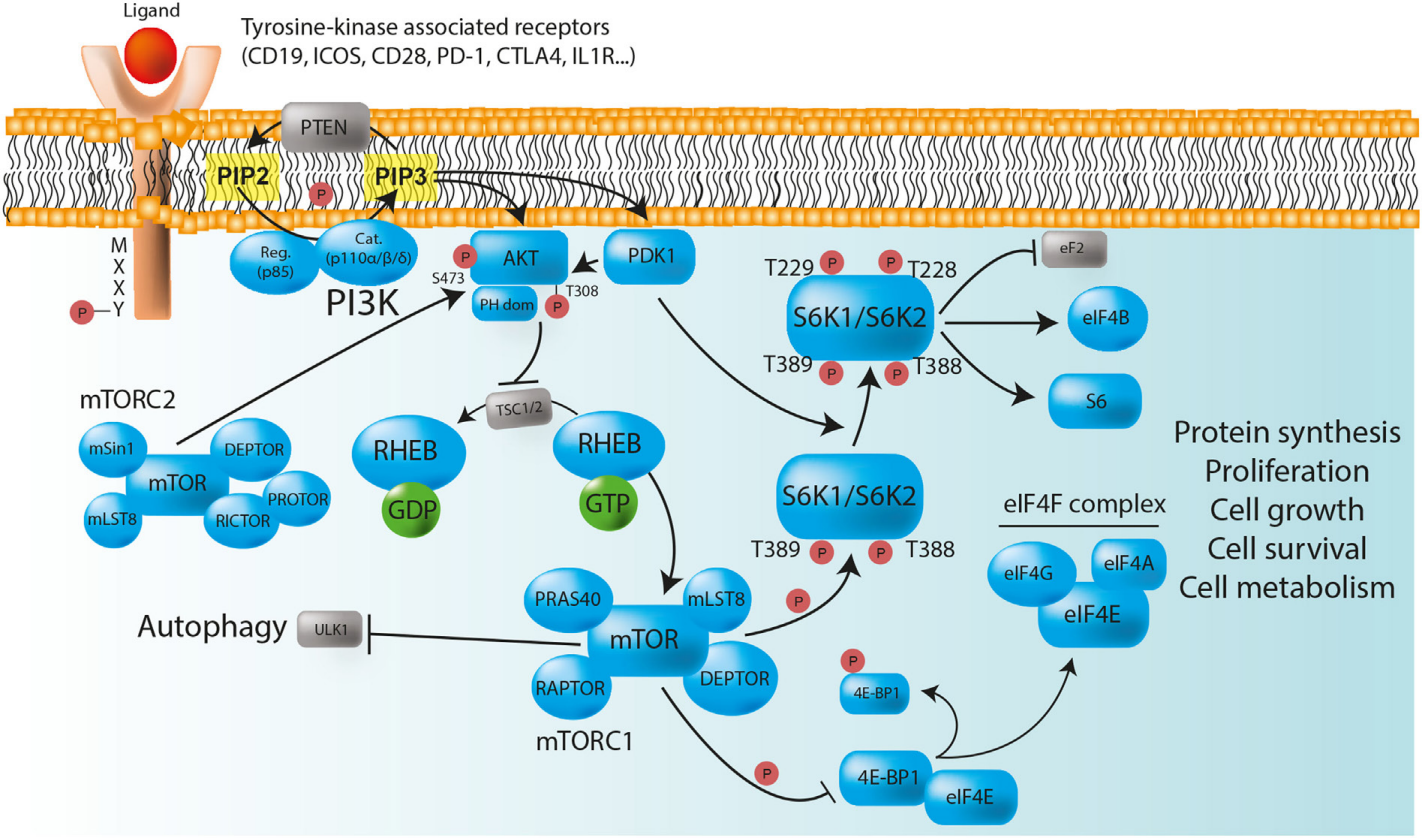
The PI3K/AKT/mTOR/S6K pathway plays a major role in the control of immune cell homeostasis. Class IA PI3Ks are heterodimeric molecules composed of a p110 catalytic subunit (p110α, p110β, or p110δ) and a p85 regulatory subunit. In immune cells, class IA PI3Ks can be activated *via* multiple surface tyrosine kinase-associated receptors [e.g., BCR, TCR, TLR, CD19, ICOS, PD-1, and CTLA-4] that bear YXXM motifs in their cytoplasmic domain. In the absence of ligand binding, the TSC1/TSC2 complex negatively regulates mTORC1, and therefore protein synthesis, by converting RHEB into its inactive GDP-bound state. After receptor activation, phosphorylated YXXM motifs provide binding sites for the p85 regulatory subunit that brings the p110 catalytic subunit to the membrane, where it converts PIP_2_ to PIP_3_. PIP_3_ serves as plasma membrane docking sites for PH-domain containing proteins, such as AKT, and its upstream activator PDK1. The activity of AKT is also positively regulated by mTORC2. Once phosphorylated, AKT inhibits the TSC1/TSC2 complex, and allows the release of GTP-bound RHEB, thereby enabling mTORC1 activation. Activated mTORC1 triggers biosynthetic pathways (protein synthesis) essential for cell proliferation, survival, and metabolism through S6Ks and 4E-BP1 phosphorylation, while inhibiting ULK1, and therefore autophagy. S6K phosphorylate numerous substrate, including ribosomal protein S6, eukaryotic translation initiation factor eIF4B, and eukaryotic elongation factor 2 (eEF2) kinase. The phosphorylation of 4E-BP1 prevents its binding to the cap-binding protein eIF4E, allowing it to participate in the formation of the eIF4F complex, which is composed of the DEAD-box RNA helicase eIF4A, the cap-binding protein eIF4E, and the large “scaffold” protein eIF4G, and which is required for the initiation of cap-dependent translation. PTEN is a negative regulator of PI3K/AKT/mTOR/S6K signaling pathway that dephosphorylates PIP_3_ back to PIP_2_. Red circles: phosphorylation; normal arrows: activation; blunt arrows: inhibition.

S6 kinase activation involves a complex signaling cascade that connects a number of critical kinases, including PI3Ks, AKT (also called PKB for protein kinase B), and mTOR (11) (Figure 1). The PI3K/AKT/mTOR/S6K pathway plays a major role in the control of cell proliferation (increase in number), cell growth (increase in size), survival, and metabolism (12). It is therefore crucial in the regulation of immune responses, as well as in the promotion of B cells, T cells, and myeloid cells differentiation, activation, and function (9).

Among the different classes of PI3Ks, class IA molecules have the most important function in immune cells (13). Those heterodimeric proteins are formed by the association of a catalytic subunit of approximately 110 kDa (p110α, p110β, or p110δ encoded by *PIK3CA, PIK3CB*, and *PIK3CD* respectively), and a Src-homology 2 (SH2) domain-containing regulatory subunit (p85, p50, and p55α encoded by *PIK3R1*; p85β encoded by *PIK3R2*; and p55γ encoded by *PIK3R3*). The catalytic subunits p110α and p110β are widely expressed, whereas the expression of p110δ is restricted to leukocytes (13, 14). The regulatory subunit controls the cellular location and the activity of the enzyme by recruiting the catalytic subunit to membrane-associated proteins that have been phosphorylated on YXXM motifs by tyrosine kinases (12, 13). In immune cells, class IA PI3Ks can be activated *via* multiple surface tyrosine-kinase-associated receptors, including the T- and B-cell receptors (TCR and BCR, respectively), toll-like receptors (TLRs), as well as various co-receptors [CD19, inducible T-cell costimulator (ICOS), CD28, PD-1, and cytotoxic T-lymphocyte-associated protein 4 (CTLA-4)], and cytokine receptors (IL-1, IL-2, IL-4, IL-12, and IFN-γ) that contain YXXM motifs in their cytoplasmic domain (12). After activation, class I PI3Ks catalyze the conversion of phosphatidylinositol-(4,5)-bisphosphate [PI(4,5)P_2_ or PIP_2_] to phosphatidylinositol-(3,4,5)-trisphosphate [PI(3,4,5)P_3_ or PIP_3_] (12). PIP_3_ acts as binding sites for various intracellular enzymes harboring pleckstrin-homology (PH) domains, in particular for the serine/threonine kinase AKT, which is then recruited at the inner leaflet of the cell membrane to be phosphorylated. The activity of AKT is positively regulated by the binding of PIP_3_ to its PH domain, but also by the phosphorylation at position Thr308 by phosphoinositide-dependent kinase-1 (PDK1) and at position Ser473 by mTORC2 (15) (Figure 1). Once AKT is activated, it inhibits the tuberous sclerosis heterodimeric complex (TSC1/TSC2 complex), inducing the release of the GTP-binding protein Ras homolog enriched in brain (RHEB) from the inhibition by TSC2, therefore enabling the activation of mTORC1 (16) (Figure 1).

The serine/threonine kinase mTOR was identified while investigating the mechanism of action of rapamycin (also known as sirolimus), an immunosuppressive drug inhibiting mTOR enzymatic activity that is currently used to prevent organ transplant rejection and to treat lymphoproliferative diseases (17, 18). mTOR associates with distinct sets of proteins to form the intracellular signaling complexes mTORC1 and mTORC2 (8). Both complexes contain mammalian lethal with SEC13 protein 8/G protein β subunit-like (mLST8/GβL) and DEP domain-containing mTOR interacting protein (DEPTOR). In contrast, the partners regulatory-associated protein of mTOR (RAPTOR) and proline-rich AKT substrate 40 kDa (PRAS40) define the mTORC1 network, whereas rapamycin-insensitive companion of mTOR (RICTOR), stress-activated map kinase-interacting protein 1 (mSIN1), and protein observed with Rictor (PROTOR) are specific to the mTORC2 complex (8, 19, 20) (Figure 1). The major function of mTORC1 is to sense nutrients and mitogenic signals (8, 19, 20). Thus, when conditions are favorable, mTORC1 triggers biosynthetic pathways essential for cell growth and proliferation, mainly through direct phosphorylation of ribosomal S6K and eukaryotic translation initiation factor 4E (eIF4E) binding protein 1 (4E-BP1) (8, 19, 20). mTORC1 also inhibits the serine/threonine kinase ULK1, thereby suppressing autophagy, a conserved catabolic process by which double-membrane vesicles (autophagosomes) engulf cytoplasmic contents for lysosomal degradation. Autophagy allows the recycling of cellular components and the generation of nutrients under metabolic stress, promoting cell survival (8, 21, 22). It is also implicated in more complex functions and participates in the regulation of immunity (23). Overall, the phosphorylation of S6Ks and 4E-BP1, along with the suppression of autophagy by active mTORC1, are essential for cell growth (24). Conversely, in case of starvation, AMP-activated protein kinase (AMPK) inactivates mTORC1 and phosphorylates the active sites of ULK1, therefore, enabling autophagy initiation (8, 21, 22). mTORC2 plays various roles in cell survival, metabolism, proliferation, and cytoskeleton organization *via* the phosphorylation of AKT on Ser473 (mTORC2-dependent), leading to the phosphorylation, sequestration, and further inhibition of Forkhead box protein O (FOXO) (9). Negative regulators controlling PI3K/AKT/mTOR/S6K pathway include the phosphatase and tensin homolog (PTEN) that dephosphorylates PIP_3_ back to PIP_2_, thereby downregulating AKT signaling (25) (Figure 1).

Together with 4E-BP1, ribosomal S6Ks represent the best characterized substrates of mTORC1 (11, 26, 27). Like AKT, S6K1 (isoforms p70- and p85-S6K1), and S6K2 (isoforms p54- and p60-S6K2) belong to the AGC serine/threonine kinases family (26). The S6K activation begins with the phosphorylation of serine residues in the C-terminal domain that expose the internal region of the protein, allowing mTOR to phosphorylate Thr389 in S6K1 and Thr388 in S6K2. Indeed, S6K activation absolutely requires mTORC1-mediated phosphorylation (28). The subsequent phosphorylation by PDK1 at Thr229 in S6K1 and at Thr228 in S6K2 leads to their full activation (26) (Figure 1). S6K proteins originally gained their name due to their ability to phosphorylate ribosomal protein S6, a component of the 40S ribosome subunit, and their preferred phosphorylation motif has been characterized as RXRXXS/T (26). S6K1 and S6K2 have many functional similarities. They regulate several cellular and molecular processes, including transcription, protein synthesis, metabolism, cell proliferation, and survival (11, 26, 28). Although S6K1 has been more extensively studied, some distinct functions of S6K2 have been described (29). For instance, it has been shown that S6K2 plays a role in Th17 differentiation through the regulation of the transcription factor RORγ (30) despite a more recent study suggesting that this function may be context-specific (31). Ribosomal protein S6 was the first discovered substrate of S6Ks. It promotes biosynthetic pathways that are important for cell growth (27, 28), but the functional significance of its phosphorylation still remains not fully understood (28). However, the analysis of the phosphorylation status of p70-S6K1 (at Thr 389) and its substrate ribosomal protein S6 (at Ser240/244; S6K dependent) is widely and routinely used as a readout of mTORC1 activity (32, 33), in particular in lymphocytes populations, where other mTOR signaling markers are more difficult to monitor. A number of other S6K1 substrates have been involved in the regulation of protein synthesis at levels of initiation (eIF4B: eukaryotic translation initiation factor 4B), and elongation (eEF2: eukaryotic elongation factor 2), but also in RNA splicing (CBC: cap binding complex; SKAR: S6K1 Aly/REF-like target) (Figure 1). In addition, S6K1 plays a role in cell survival by blocking apoptosis through phosphorylation of the pro-apoptotic protein Bcl-2-associated death promoter (BAD), thereby preventing its interaction with BCL-X or BCL-2 (11, 26, 28). Some evidences also indicate that S6K1 may participate in cytoskeleton dynamics, in particular in F-actin reorganization (34).

Studies in animal models have suggested that reduced PI3K/AKT/mTOR/S6K signaling (hypoactivation) can lead to immune deficiency, whereas uncontrolled PI3K/AKT/mTOR/S6K signaling (hyperactivation) is associated with autoimmunity and hematological malignancies (12). Nevertheless, this simplistic dichotomous model does not reflect the highly complex regulation of this pathway. Indeed, several human PIDs that are associated with a hyperactivation of the PI3K/AKT/mTOR/S6K pathway have features of both immunodeficiency and immune dysregulation, suggesting a tight and dynamic modulation of the signaling cascade for optimal immune cell function.

mTOR plays a central role in the regulation of immune responses evidenced in numerous studies showing that mTOR or mTORC1 inhibition can have both positive and negative effects on lymphocytes, in particular on T-cell development and functions [reviewed in Ref. (9)]. The mTOR hypomorphic mouse, which is a model of mTORC1/mTORC2 inhibition [murine *Mtor* knockout (KO) is lethal and there are no reported cases of human loss-of-function (LOF) mutations in *MTOR*] is characterized by an immunodeficient phenotype with impaired development, proliferation, and migration of lymphocytes, as well as abnormal antibody production (35). Reduced mTOR expression results in decreased phosphorylation of the mTORC1 target p70-S6K1 and of the mTORC2 target AKT (phosphorylation at Ser473) in fibroblasts and TCR stimulated T cells. However, despite reduction of p70-S6K1 phosphorylation in murine B cells activates with lipopolysaccharide (LPS), mTORC2 activity is increased, suggesting that AKT regulation may be cell-type specific (35). In addition, PI3K/AKT/mTOR pathway seems to play differing roles during the differentiation and function of regulatory T cells (Tregs). Tissue tolerance is associated with the upregulation of enzymes that consume many of the essential amino acids (36). These starvation conditions lead to mTOR inhibition, promoting the expression of FoxP3 in naïve T cells, and therefore the generation of CD4^+^ FoxP3^+^ Tregs (37). In fact, continued TCR signaling and constitutive PI3K/AKT/mTOR activity antagonizes Foxp3 induction (9, 37, 38). However, under mTOR inhibitory conditions, Tregs are not optimally functional, requiring mTOR re-activation or inflammatory conditions to acquire their full suppressive potential. Alternate cycles of mTOR activity may therefore be needed for optimal functional induction of Tregs (37, 39). The mTOR downstream effectors S6Ks are essential in controlling the cell size and proliferation of certain cell types such as hepatocytes (40, 41). However, in contrast to mTOR, the functions of S6K1 and S6K2 in lymphocytes still remain controversial (33). Simultaneous deletion of *S6K1* and *S6K2* genes in a murine model was associated with a severe reduction in viability due to perinatal lethality, but single *S6K1* or *S6K2* KO mice did not exhibit obvious immune defects (although no detailed immunological study was performed) (41, 42). In addition, it has been shown *in vitro* using *S6K1/S6K2* double KO T and B cells that S6K activity is dispensable for lymphocytes growth and proliferation after antigen receptor engagement (33). Germline deletion of *Rps6* that encodes for ribosomal protein S6 is embryonically lethal (43) and T cell-specific deletion of *Rps6* abolishes thymic T-cell development (44). By contrast, the role of S6 phosphorylation is not well understood. Knockin mice in which all serine residues of S6 protein have been mutated to alanine to prevent phosphorylation by S6Ks are viable (45) and show normal T-cell activation and differentiation (46). All these data clearly demonstrate the complexity of PI3K/AKT/mTOR/S6K pathway regulation.

## Gain-of-Function (GOF) Mutations in the Genes Encoding Class I PI3K Cause Activated PI3Kδ Syndrome (APDS)

Hyperactivation of the PI3K/AKT/mTOR/S6K signaling pathway in immune cells can be the consequence of heterozygous GOF mutations in the genes encoding for PI3Kδ that cause an immune dysregulation disorder called activated PI3Kδ syndrome [APDS; also known as “p110δ activating mutation causing senescent T cells, lymphadenopathy, and immunodeficiency” (PASLI)] (47). Molecularly, APDS encompasses two different disorders: APDS1 and APDS2. APDS1 (or PASLI-CD) is the consequence of mutations in the *PIK3CD* gene encoding for p110δ, the catalytic subunit of PI3Kδ that result in single-amino-acid substitutions leading to p110δ overactivation. APDS2 (or PASLI-R1) results from mutations in the *PIK3R1* gene encoding for p85α, the regulatory subunit of PI3Kδ. These mutations impair the binding of p85α to its cognate partner p110δ that is, therefore, inefficiently inhibited (47–51). Up to date, more than 150 APDS patients have been reported (48–68). They display features of both immune deficiency and immune dysregulation, and all of them present with early-onset, as well as severe and recurrent sino-pulmonary infections, mostly by encapsulated bacteria (47, 58). Benign lymphoproliferation (hepatosplenomegaly, lymphadenopathy, focal nodular lymphoid hyperplasia), various autoimmune manifestations, and B cell lymphomas are also frequently observed (47, 54, 55, 58, 61). Growth retardation is, however, commonly associated with APDS2, but not APDS1 (58, 66).

Most APDS patients have elevated transitional B cells, reduced class-switched memory B cells, variable immunoglobulin levels (mainly reduced IgG and increased IgM levels, hypogammaglobulinemia, or in some cases agammaglobulinemia) associated with a poor vaccine response, and an impaired *in vitro* B cell isotype switching (47, 51, 64, 69). Abnormalities in B lymphocytes from APDS patients recapitulate the defects of class-switch recombination that are observed in B lymphocytes from PTEN-deficient mice (70). Although APDS was initially described as a common variable immunodeficiency (CVID)-like disease, affected patients also suffer from recurrent herpes virus infections (i.e., EBV, CMV, and VZV), indicating an impaired T cell function (47, 52, 54, 56–58, 65). In addition, the majority of APDS patients show a progressive CD4^+^ T cell lymphopenia with a decreased frequency of CD4^+^ naïve T cells [in contrast to the lethal CD4^+^ T cell hyperplasia that is described in mice with a T cell-specific deletion of *PTEN* (71)], but an excessive accumulation of terminally differentiated, senescent CD8^+^ effector T cells (64). Considering the T cell abnormalities, APDS may be classified as combined immunodeficiency (CID) rather than as CVID-like disease.

In T cells, PI3Kδ is activated downstream of CD28, leading to enhanced AKT and mTOR signaling, which blocks autophagy but stimulates T cell proliferation and terminal differentiation through the phosphorylation of S6K (12). Activated AKT also mediates the phosphorylation and subsequent degradation of FOXO transcription factors that regulate T cell expansion and memory T cell differentiation (72). The analysis of PI3K signaling in T cells from APDS patients showed a constitutive hyperphosphorylation of both AKT (on Thr308: PI3K/PDK1 dependent and on Ser473: mTORC2 dependent) and S6 (on Ser235/236 and Ser240/244: mTORC1 dependent) (50–52, 64, 65, 67). The general overactivation of the PI3K/mTOR/S6K signaling pathway promotes the switch to an anabolic cellular state with increased aerobic glycolysis that is required for the expansion of effector T cells (73). Downregulation of mTOR signaling and reversion to a catabolic cellular state by autophagy induction, are, however, crucial for memory T cell formation and prolonged survival (73). In APDS patients, the constant maintenance of aerobic glycosis restrains the function and survival of memory CD8^+^ T cells, leading to an abundance of senescent effector and short-lived effector memory CD8^+^ T cells that exhibit a poor recall response *in vitro* and could account for the defective antiviral immunity *in vivo* (64, 65, 74). Similarly, high AKT and S6 phosphorylation levels were observed in transformed EBV-B cells, peripheral blood mononuclear cells, and isolated B cells (total B cells and isolated B cell subsets) from APDS patients at basal state and after B cell stimulation (48, 51, 52, 65). However, the link between the increased PI3K/mTOR/S6K signaling in B cells and the observed B cell phenotype is still a focus of research.

The insights into the pathophysiology of APDS allowed refining the therapeutic approaches. Indeed, it has been shown that *in vitro* treatment of unstimulated T cell blasts with the mTOR inhibitor rapamycin (sirolimus) leads to a decrease of S6 hyperphosphorylation (64). More notably, the administration of rapamycin was found to improve the clinical and immunological phenotype of two APDS patients with a reduction of hepatosplenomegaly and lymphadenopathy, as well as a normalization of T cell subpopulations (64, 67). However, PI3Kδ regulates additional pathways to mTOR (such as FOXO for example) and mTOR is also controlled by PI3K-independent pathways (13). Therefore, selective inhibitors of the PI3Kδ subunit, which have already shown remarkable success in certain hematologic malignancies, should be considered as future therapeutic options in APDS patients. Both *in vitro* and *in vivo* data support the specific inhibition of PI3Kδ as a promising therapy. Indeed, the selective p110δ inhibitor IC87114 is able to dampen the activity of the mutated PI3Kδ *in vitro* in APDS1 patients’ T cells (52), and both p110δ (APDS1) and p85α (APDS2) are strongly inhibited *in vitro* by the PI3Kδ-specific inhibitor idelalisib (GS-1101 or CAL-101), which is currently approved by the US-Food and Drug Administration for the treatment of chronic lymphocytic leukemia (50, 75). In addition, the first clinical trial (#NCT02435173) that has been conducted by Novartis with the PI3Kδ-specific inhibitor leniolisib (CDZ173) in six APDS patients produced encouraging results (76). Oral administration of leniolisib during 12 weeks was well tolerated and was associated with an improvement of both laboratory and clinical parameters (reduction of peripheral transitional B cells, naive B cells, and senescent T cells; decrease of IgM and inflammatory cytokines levels; reduction of splenomegaly and lymphadenopathy) (76). Another clinical trial for an inhaled PI3Kδ inhibitor, sponsored by GlaxoSmithKline, is currently ongoing in patients with APDS (#NCT02593539) (47).

## LOF Mutations in *PTEN* Lead to an Activated PIK3δ Syndrome-Like Deficiency (APDS-Like)

*PTEN* encodes a lipid and protein phosphatase that dephosphorylates PIP_3_ back to PIP_2_ (77), thereby inhibiting the PI3K/mTOR/AKT/S6K signaling cascade (25). Impairment of PTEN activity is associated with an overabundance of PIP_3_ and a constitutive downstream activation of AKT, leading to cellular proliferation and overgrowth (78).

A complete disruption of *Pten* in mouse results in early embryonic death (79), whereas *Pten* heterozygous mutant mice display hyperplastic-dysplastic features, develop spontaneously tumors (80), and present a lethal polyclonal autoimmune disorder with a phenotype that is reminiscent of *Fas*-deficient mice (81). Mice carrying a B cell-specific deletion of *Pten* show abnormal B cell differentiation and function, with increased numbers of marginal zone and B1-a B cells in the spleen, a production of serum autoantibodies, an impaired response to T-dependent and T-independent immunizations, as well as a defect in immunoglobulin class-switch recombination (70, 82, 83).

In humans, heterozygous germline mutations in *PTEN* may cause different autosomal dominant disorders including Cowden syndrome (CWS; OMIM 158350), Bannayan–Riley–Ruvalcaba syndrome (OMIM 153480), and Proteus syndrome (OMIM 176920), which are characterized by the development of multiple benign hamartoma and malignant tumors (84–86). The term *PTEN* hamartoma tumor syndrome (PHTS) is therefore used to describe any patient with a germline *PTEN* mutation regardless of the phenotype (78). Browning et al. reported a case of CWS associated with CID (87). In line with this observation, recent studies indicated that heterozygous LOF mutations in *PTEN* lead to immunodeficiency and immune dysregulation, with a clinical and immunological presentation that resembles APDS phenotype, including recurrent infections, organomegaly, and CD4^+^ T cell lymphopenia (68, 88). However, immunodeficiency seems to occur only in some, but not all, patients with *PTEN* LOF mutations (68). Similarly to patients with heterozygous GOF mutations in *PIK3CD, PTEN* mutations are associated with an aberrant hyperactivation of the PI3K/AKT/mTOR/S6K pathway with increased phosphorylation of AKT, mTOR, and S6 in T cells (68, 87). Driessen et al. further studied, in a cohort of nine PHTS patients, the impact of germline *PTEN* mutations on the peripheral B cell development and the humoral immune response (89). They observed decreased counts of switched memory B cells associated with a dysregulated T-dependent B cell response, abnormalities in class-switch recombination, and decreased somatic hypermutation, resulting in hypogammaglobulinemia in about one-third of the patients (89). In mice, it has been shown that the level of activation-induced cytidine deaminase, the main regulator of somatic hypermutation and class-switch recombination, is regulated by the PI3K/AKT signaling cascade (70, 83, 90). This could explain, at least in part, the dysregulated humoral immune response observed in human PTEN deficiency (89).

Surprisingly, despite PTEN dysfunction, PHTS patients display a normal frequency and phenotype of CD4^+^ FoxP3^+^ Tregs, as well as a normal activation of the downstream signaling pathway with similar percentages of S6-phosphorylated Tregs in PHTS patients and controls subjects (88). In this cell subset, the enzyme PH domain leucine-rich repeat protein phosphatase (PHLPP), located downstream of PTEN and highly expressed in normal Tregs, provides a complementary phosphatase activity that is important for limiting PI3K hyperactivation (88). *PTEN* haploinsufficiency leads to APDS-like immune dysregulation, but the compensatory activity of the phosphatase PHLPP may help to maintain checkpoint control at the immunological synapse in human Tregs (88), possibly preventing the development of autoimmune manifestations.

## Lipopolysaccharide-Responsive Beige-Like Anchor Protein (LRBA) Deficiency is Associated with Impaired mTOR/S6K Signaling in T Cells

Lipopolysaccharide-responsive beige-like anchor protein (LRBA) belongs to the Beige and Chediak-Higashi (BEACH) domain-containing protein (BDCP) family together with eight other human proteins (91, 92). Although the exact functions of BDCPs remain unclear, they are considered to act as scaffolding molecules forming multiprotein complexes involved in vesicle trafficking and receptor signaling (92). Biallelic mutations in *LRBA* cause a PID and immune dysregulation disorder known as LRBA deficiency (93). LRBA-deficient patients show an early-onset broad spectrum of clinical and immunological manifestations, including recurrent infections, organomegaly, inflammatory bowel-like disease, hypogammaglobulinemia, and autoimmunity (94, 95). Several LRBA-deficient patients present with an immune dysregulation, polyendocrinopathy, enteropathy, X-linked (IPEX)-like syndrome, indicating Treg cells impairment, that might contribute to the development of the various autoimmune manifestations (96). In fact, nearly two-thirds of LRBA-deficient patients have reduced Tregs frequency (95) with decreased expression of the canonical Treg markers (FOXP3, CD25, Helios, CTLA-4) and impaired Treg cell-mediated suppression (96). Additional perturbations observed in the T cell compartment such as increased proportion of circulating follicular helper T cells (T_FH_) and decreased proportion of circulating follicular Tregs suggest an ineffective regulation of autoantibodies’ production (96). Although the frequency of recent thymic emigrants seems to be normal, conventional T cells and Tregs from LRBA-deficient patients exhibit an increased apoptosis (96). In mice, Treg-specific disruption of mTORC1 (through the deletion of *Raptor*) leads to a profound loss of Treg suppressive activity with early development of a lethal autoimmunity and lymphoproliferation (39). Mechanistically, mTORC1 signaling promotes the cholesterol/lipid metabolism that is crucial for cell proliferation and for CTLA-4 upregulation, thereby establishing functional Treg competency (39). CTLA-4 belongs to the T cell co-stimulatory molecule family, including CD28, ICOS, and PD1. It is a critical negative regulator of T cell proliferation that serves as a “checkpoint” of immune responses (97). Interestingly, the role of LRBA in CTLA-4 post-transcriptional regulation is currently the only proven cellular function for LRBA (98). Specifically, LRBA binds through its BEACH domain to the cytoplasmic tail of CTLA-4, allowing its vesicular transport to the plasma membrane of Tregs, and activated conventional T cells (98). CTLA-4 is then able to remove, *via* transendocytosis, the CD80 and CD86 co-stimulatory molecules from the cell surface of antigen-presenting cells, thereby controlling T cell activation (99). However, when LRBA is absent, the adaptor protein AP-1 binds to CTLA-4, leading to its lysosomal degradation (98). Decreased CTLA-4 expression might therefore contribute to the high frequency of autoimmune manifestations observed in patients with LRBA deficiency (94, 95). Indeed, patients with heterozygous LOF mutations in *CTLA-4* develop an immune dysregulation syndrome with an LRBA-deficiency-like clinical phenotype (100–102) known since 2014 as CTLA-4 deficiency. Surprisingly, CTLA-4 was assessed to bind to PI3K with the same avidity as CD28, possibly leading to the activation of PDK1 that phosphorylates AKT at position Thr308 (103, 104), thereby activating mTORC1 signaling cascade. Moreover, in T cells, CTLA-4 dependent activation of PI3K and AKT was shown to sustain T cell anergy without cell death (105). However, the intracellular signaling capacity of CTLA-4 was recently questioned (106). In contrast, it has been reported that activated LRBA-deficient CD4^+^ and CD8^+^ T cell subsets show an impaired mTORC1 and mTORC2 activity with a reduced phosphorylation of downstream mTORC1 (S6 and 4E-BP1) and mTORC2 (AKT at position Ser473) substrates (96). Therefore, the PI3K/mTOR/S6K signaling pathway should also be investigated in patients with CTLA-4 deficiency.

Besides Tregs dysfunction, patients with LRBA deficiency present defects in the B cell compartment with reduced numbers of switched memory B cells and plasmablasts, impaired immunoglobulin secretion, low proliferative responses, and a high susceptibility to apoptosis (95, 96). In addition, LRBA-deficient B cells show an impairment of the autophagic flux with an abnormal accumulation of autophagosomes (93). Pengo et al. have shown that autophagy is required for plasma cell homeostasis and long-lived humoral immunity by limiting endoplasmic reticulum stress and immunoglobulin synthesis, while sustaining energy metabolism and plasma cell viability (107). The impaired B cell differentiation and hypogammaglobulinemia observed in LRBA-deficient individuals may therefore be attributable to an increased B cell apoptosis and a reduced plasma cell survival due to defective autophagy. In fact, autophagy is also essential for the survival of memory B cells, and for the maintenance of protective antibody responses required to control viral infections in mice (108). In addition, the accumulation of apoptotic cells may trigger as well the development of autoimmunity (109). mTOR plays a key role at the interface of the pathways controlling cell growth and autophagy. Under nutrient starvation, reduced growth factor signaling, or stress conditions, mTOR is inhibited, and autophagy is therefore promoted. Conversely, the activation of the PI3K/AKT pathway negatively regulates autophagy induction (22). It has been previously suggested that LRBA might act as a scaffold protein, coordinating the assembly and activation of mTOR complexes or of protein networks involved in the autophagic process, as well as the recruitment of downstream molecules (96). Future studies addressing mTOR/S6K signaling in the B cell compartment of LRBA-deficient patients may help to further clarify the links between LRBA, autophagy, and B cell homeostasis.

## Mutations Affecting the CARD11-BCL10-MALT1 (CBM) Signalosome Complex are Responsible for Novel PID Phenotypes with an Abnormal Activation of the mTOR/S6K Signaling Pathway

Upon TCR and CD28 activation, the adapter protein caspase recruitment domain-containing protein 11 (CARD11, also called CARMA1), which is specifically expressed in hematopoietic cells, becomes phosphorylated by protein kinase C and other kinases including AKT (110). Phosphorylated CARD11 recruits B-cell lymphoma/leukemia 10 (BCL10) and mucosa-associated lymphoid tissue lymphoma translocation protein 1 (MALT1) to form a scaffold called the CBM (CARD11-BCL10-MALT1) signalosome complex that is necessary for optimal activation of the canonical nuclear factorp-κB (NF-κB) pathway (111). Recently, it has been shown that CARD11 and the paracaspase MALT1, but not BCL10, are also required for an optimal activation of the mTOR/S6K pathway in T cells in response to TCR and CD28 co-receptor stimulation (112).

LOF autosomal recessive mutations in *CARD11, MALT1*, and *BCL10* are the cause of a new group of CIDs characterized by recurrent sinopulmonary infections, dysregulated B cell development, and abnormal T cell proliferation despite normal lymphocytes counts, due to a defective canonical NF-κB activation after antigen receptor stimulation (113–118). However, these recently described disorders have a distinct phenotype from other known PIDs affecting the NF-κB axis (113). In addition, there are notable differences between the clinical presentation of CARD11, MALT1, and BCL10 deficiencies (113). For instance, CARD11-deficient patients display variable immunoglobulin levels and Tregs numbers, a predominance of *Pneumocystis jirovecii* infections, but no gastrointestinal inflammation, whereas BCL10 deficiency has an impact on lymphocytes (low memory T cells) and fibroblasts but not on myeloid cells. CARD11 and BCL10 deficiencies are both characterized by the lack of autoimmune manifestations despite reduced Treg numbers, possibly reflecting the individual nuanced and independent functions of the CBM proteins (113). CARD11-deficient and *MALT1*-knockdown cells are characterized by a reduced phosphorylation of S6K and S6, emphasizing the role of CARD11 and MALT1 in the mTOR/S6K signaling pathway (112). In addition, the metabolic reprogramming and the proliferation of CD4^+^ T cells that are also mTORC1 dependent are impaired after MALT1 inhibition (112).

Very recently, Ma et al. have described rare heterozygous hypomorphic *CARD11* mutations in eight individuals from four unrelated families with severe atopic dermatitis (119). The phenotype also included variable cutaneous and respiratory infections (88%), eosinophilia (86%), B cell lymphopenia (29%) with low IgM, but normal or elevated IgA (43%), and hyper-IgE (71%) (119). Transfection of mutant CARD11 constructs into T cell lines demonstrated both LOF and a dominant-negative effect on mTORC1 (indicated by reduced S6 phosphorylation), but also on NF-κB signaling, at basal state and after antigen-receptor-induced stimulation. Similarly, mTORC1 activity was also attenuated in T cells, and to a lesser extent in B cells, from patients with heterozygous hypomorphic mutations in *CARD11*, whereas AKT phosphorylation on Ser473 (mTORC2-dependent) was normal (119). mTOR activity is known to be crucial for T helper (T_H_) cell differentiation (120). Patients’ T cells were characterized by an impaired T_H_1 cytokine production (low IFN-γ) and a T_H_2-skewed phenotype, consistent with their atopic predisposition. The reduced CARD11-dependent mTORC1 activation could contribute to impaired T_H_1 differentiation in these patients, allowing mTORC2-dependent T_H_2 response to dominate (119). The role of CARD11 in the regulation of mTORC1 activation depends on its ability to facilitate TCR-induced upregulation, but also on its capacity to activate sodium-dependent neutral amino acid transporter type 2 (ASCT2, also known as SLC1A5), an essential amino acid transporter required for extracellular glutamine import during T cell activation (121). Indeed, T cells from patients with germline hypomorphic *CARD11* mutations showed reduced ASCT2 upregulation after TCR activation (119). However, the addition of exogenous glutamine in T cell culture medium was able to boost mTORC1 activation with increased S6 phosphorylation, and to partially correct the T_H_1 cell defect including proliferation and IFN-γ production (119). Further studies are required to evaluate whether glutamine supplementation, a very simple therapeutic intervention, could ameliorate atopic dermatitis in patients with *CARD11* mutations (119). This clearly illustrates that a fine comprehension of the mechanisms regulating the mTOR/S6K signaling pathway is an essential prerequisite for a proper improvement of the patients’ therapeutic management.

Germline heterozygous GOF mutations in *CARD11* have been linked to a novel congenital B cell lymphoproliferative disorder called BENTA for “B cell Expansion with NF-κB and T cell Anergy” (122, 123). Five different GOF *CARD11* mutations in 16 patients have been described so far (74, 122–124). They abrogate the requirement for antigen receptor engagement in CARD11 activation, resulting in spontaneous CBM signalosome formation, and constitutive NF-κB activation that is responsible for an excessive accumulation of both immature transitional B cells, and polyclonal mature naive B cells (122, 125). BENTA patients develop massive B cell lymphocytosis early in life accompanied by splenomegaly and lymphadenopathy, but without obvious signs of autoimmunity (122, 123). Moreover, GOF *CARD11* mutations can potentially predispose to B cell malignancies (74, 122, 126). Despite excessive B cell accumulation, BENTA disease is associated with an underlying immunodeficiency characterized by low frequencies of circulating memory and class-switched B cells, poor humoral response to T cell-independent polysaccharide-based vaccines, impaired plasma cell differentiation, and low IgM as well as variable IgA/IgG secretion. Recurrent sinopulmonary infections are common, and opportunistic viral infections have been noted in some patients (74). Although circulating T cells are present at normal numbers, they are hyporesponsive upon *in vitro* stimulation, suggesting that they may be anergic (74, 113, 122–124). GOF mutations in *CARD11* affect B and T cells differently, promoting proliferation and survival of B lymphocytes *versus* anergy in T lymphocytes, but the underlying mechanisms remain poorly understood (74). Similarly to LOF *CARD11* mutations, BENTA-associated mutations may therefore perturb other CARD11-dependent downstream signaling cascades including the mTOR/S6K pathway (74). However, to our knowledge, there are currently no published data on mTOR and S6K phosphorylation in the context of BENTA disease.

## Future Studies Should Explore mTOR/S6K Signaling Pathway in T Cells from CARMIL2-Deficient Patients

Biallelic LOF mutations in the gene encoding for the cell membrane-cytoskeleton-associated protein RLTPR (RGD, leucine-rich repeat, tropomodulin and proline-rich-containing protein), also known as CARMIL2 (capping protein regulator and myosin 1 linker 2), have been shown to be responsible for a novel PID disorder characterized by cutaneous and pulmonary allergy, by various bacterial, fungal, and mycobacterial infections, as well as by EBV lymphoproliferation (EBV^+^ smooth muscle tumors) (127, 128). In addition to its involvement in cell polarity and migration (129), CARMIL2 plays an important role in T cells by acting as a scaffold protein, bridging CD28 to CARD11 and therefore to the NF-κB signaling axis (130). Mutations in *CARMIL2* prevent the association of CARMIL2 with CARD11 (130). CARMIL2-deficient T cells have a perturbed cytoskeletal organization leading to abnormalities in T cell polarity and migration, but also an impaired CD28-mediated co-signaling with a defective activation of the canonical NF-κB pathway (127, 128, 130). CARMIL2-deficient patients have a normal production of T_H_2 cytokines, but a reduced secretion of T_H_1, as well as T_H_17 effector cytokines, and therefore the strong decrease in Treg numbers does not result in the development of autoimmunity (127, 130). This phenotype is reminiscent of *CARD11*-deficient patients (119). Considering the newly described role of CARD11 in the mTOR/S6K pathway activation following TCR and CD28 stimulation, future studies should also address this signaling cascade in T cells from CARMIL2-deficient patients.

## Mutations in Genes Encoding for the CD19-Complex Could be Associated with a Disturbed PI3K/mTOR/S6K Signaling

CD19 is a B cell lineage-specific transmembrane protein expressed from the pro B cell stage until plasma cell differentiation (131). It forms the CD19-complex together with CD21, CD81, and CD225 on the membrane of mature B cells. This complex is recruited to the BCR after ligation by complement (C3d) opsonized antigen *via* the complement receptor 2 (CR2, also known as CD21). This increases the BCR-mediated signal into B cells, as the BCR itself only delivers a weak tonic signal. CD19, with its many tyrosine residues, amplifies this signal to properly activate B cells (131–133). Biallelic mutations in *CD19*, leading to loss of CD19 membrane expression, to concomitant reduction of CD21 levels, and hence B cell activation, have been described in CVID patients (134–137). Affected patients have recurrent bacterial infections, hypogammaglobulinemia, decreased memory B cell numbers, defective antibody response after vaccination, as well as impaired somatic hypermutation, class-switch recombination, and immunoglobulin repertoire selection (134–138). As expected, they show neither T cell defects nor signs of lymphoproliferation (134–137). However, autoimmune manifestations (thrombocytopenia, glomerulonephritis) and autoantibody production have been reported (134, 135, 137, 139). Since CD81 is required for CD19 expression on the plasma membrane, patients with CD81 deficiency display a phenotype that is highly similar to CD19-deficient patients (140, 141). Upon BCR ligation, CD19 is rapidly phosphorylated at multiple tyrosine residues, leading to the recruitment of various downstream signaling intermediates. A prominent feature of CD19 signaling is the binding of the p85α regulatory subunit and the subsequent activation of class IA PI3K, thereby promoting AKT phosphorylation (132). In the absence of CD19, AKT activity is reduced in B cells (142). However, CD19 amplifies not only BCR signaling, but also plays a crucial role in the regulation of TLR9 responses in human B cells (143). It recruits PI3K and mediates AKT as well as Bruton’s tyrosine kinase (BTK) phosphorylation after ligation of nucleic acids, controlling both early B cell activation and proliferation (143). In fact, although AKT phosphorylation at position Ser473 is still induced after BCR triggering in CD19-deficient B cells, it is strongly reduced after CpG stimulation. In addition, inhibition of PI3K and AKT results in TLR9-induced B cell activation defects that are similar to those observed in CD19-deficient B cells (143). Therefore, CD19 deficiency may also be associated with abnormal mTOR/S6K signaling in B cells, but no data are currently available in the literature. However, since the phenotype of p85α-deficient mice is much more severe than the one of CD19-deficient mice, other signaling components might compensate for the loss of CD19 (142, 144–146).

## PI3K/mTOR/S6K Signaling Should be Investigated in ICOS-Deficient Patients

Inducible T-cell costimulator (ICOS, also known as CD278) is another member of the CD28 T cell co-stimulatory molecules family (147). CD28 is expressed in resting and activated T cells, whereas ICOS expression is induced only upon T cell activation. Like CD28, ICOS delivers a positive signal that sustains T cell responses, and it is crucial for cell proliferation as well as cytokine production (148). CD28 and ICOS share a common signaling pathway, including PI3K recruitment (149, 150). In addition, ICOS plays an essential role in T_FH_ differentiation as well as in germinal center formation, and hence in isotype switching and in the development of memory B cells (151, 152). ICOS deficiency was the first monogenic defect reported to cause CVID (153). To date, homozygous mutations (deletions) in *ICOS* have been identified in 16 patients, resulting in the absence of ICOS protein on T cells (153–158). ICOS deficiency was initially considered as a “predominantly antibody deficiency” by the IUIS PID expert committee (159), but following published patients with more complex phenotypes [reviewed by Ref. (154)], allowed a reclassification of the disease as a CID (2, 3). Besides hypogammaglobulinemia (93% of the cases) associated with an increased susceptibility to bacterial infections, more than two-thirds of the patients presented with autoimmunity and immune dysregulation (mainly enteropathy and psoriasis). Viral and opportunistic infections were frequently observed, and two patients developed malignancies (154). ICOS deficiency is associated with several immunological abnormalities including decreased numbers of switched memory B cells and circulating CXCR5^+^ T_FH_ that coincide with an impaired germinal center formation (151, 154). B cell counts seem to decline progressively during the course of the disease, possibly as a consequence of a bone marrow production failure. IL-17 levels are markedly decreased in all patients who have been assessed for cytokine production, but without being associated with an increased susceptibility to *Candida* infection (154). ICOS is responsible for a greater PI3K activity than CD28, leading to a strong subsequent phosphorylation of AKT (150, 160). It bears a unique YMFM motif in its cytoplasmic tail that binds to the p85α regulatory subunit of PI3K (149).

In addition, ICOS interaction with its ligand ICOSL induces the recruitment of the PI3K regulatory subunit p50α at the synapse of T cell/antigen-presenting cells conjugates (160). ICOS deficiency should therefore be associated with impaired PI3K signaling. The activity of PI3K, as well as of downstream effector signaling molecules including mTOR and S6K, should be explored in T cells from ICOS-deficient individuals.

Regarding CD28, no PID has been associated so far with mutations in the gene encoding for this other T cell co-stimulatory receptor.

## Conclusion

There are several lines of evidence that link the PI3K/AKT/mTOR/S6K signaling pathway to PIDs. Further studies are nevertheless required to characterize more deeply the crosstalk between the PI3K/AKT/mTOR/S6K cascade and other signaling molecules, as well as the disease-specific defects. Understanding the genetics and mechanisms behind the “immune TOR-opathies” is crucial to improve the management of the patients. The use of inhibitors such as mTOR and PI3K inhibitors that specifically target this signaling pathway and could restore properly the immune function represent very promising therapeutic approaches. Selective PI3K inhibitors should be considered as future treatment options, in particular in APDS patients, with encouraging preliminary results in ongoing clinical trials.

## Author Contributions

SJ, LG-D, and BG wrote the review. MP prepared the figure. All authors concur with the submission.

## Conflict of Interest Statement

The authors declare that the research was conducted in the absence of any commercial or financial relationships that could be construed as a potential conflict of interest.

## Abbreviations

AMPK, AMP-activated protein kinase; APDS, activated PI3Kδ syndrome; ASCT2, sodium-dependent neutral amino acid transporter type 2; BAD, Bcl-2-associated death promoter; BCL10, B-cell lymphoma/leukemia 10; BCR, B-cell receptor; BDCP, BEACH domain-containing protein; BEACH, Beige and Chediak-Higashi; BENTA, B cell expansion with NF-κB and T cell anergy; BTK, Bruton’s tyrosine kinase; CARD11, caspase recruitment domain-containing protein 11; CARMIL2, capping protein regulator and myosin 1 linker 2; CBM, CARD11-BCL10-MALT1; CID, combined immunodeficiency; CMV, cytomegalovirus; CTLA-4, cytotoxic T-lymphocyte-associated protein 4; CR2, complement receptor 2; CVID, common variable immunodeficiency; CWS, Cowden syndrome; DEPTOR, DEP domain-containing mTOR interacting protein; EBV, Epstein-Barr virus; 4E-BP1, eukaryotic translation initiation factor 4E (eIF4E)-binding protein 1; FOXO, Forkhead box protein O; GOF, gain-of-function; ICOS, inducible T-cell costimulator; IPEX, immune dysregulation, polyendocrinopathy, enteropathy, X-linked; IUIS, International Union of Immunological Societies; KO, knockout; LOF, loss-of-function; LPS, lipopolysaccharide; LRBA, lipopolysaccharide-responsive beige-like anchor protein; MALT1, mucosa-associated lymphoid tissue lymphoma translocation protein 1; mLST8/GβL, mammalian lethal with SEC13 protein 8/G protein β subunit-like; mSIN, stress-activated map kinase-interacting protein 1; mTOR, mechanistic/mammalian target of rapamycin; mTORC, mTOR complex; NF-κB, nuclear factor-kappa B; PASLI, p110δ activating mutation causing senescent T cells, lymphadenopathy, and immunodeficiency; PDK1, phosphoinositide-dependent kinase-1; PH, pleckstrin-homology; PHTS, *PTEN* hamartoma tumor syndrome; PHLPP, PH domain leucine-rich repeat protein phosphatase; PID, primary immunodeficiency; PIP_2_, phosphatidylinositol-(4,5)-bisphosphate; PIP_3_, phosphatidylinositol-(3,4,5)-trisphosphate; PI3K, phosphoinositide 3-kinase; PKB, protein kinase B; PRAS40, proline-rich AKT substrate 40 kDa; PROTOR, protein observed with Rictor; PTEN, phosphatase and tensin homolog; RAPTOR, regulatory-associated protein of mTOR; RHEB, Ras homolog enriched in brain; RICTOR, rapamycin-insensitive companion of mTOR; RLTPR, RGD, leucine-rich repeat, tropomodulin and proline-rich-containing protein; S6K, S6 kinase; SH2, Src-homology 2; TCR, T-cell receptor; T_FH_, follicular helper T cells; TH, T helper; TLR, toll-like receptor; Tregs, regulatory T cells; VZV, varicella zoster virus.
